# Supplementary material for: Female peer mentors early in college have lasting positive impacts on female engineering students that persist beyond graduation
Source: Nat Commun. 2022 Nov 11;13:6837. doi: 10.1038/s41467-022-34508-x (PMC9652302; doi:10.1038/s41467-022-34508-x)
Supplement: Supplementary file 1 — Supplementary Information [file 41467_2022_34508_MOESM1_ESM.pdf]

Supplementary Materials

Subjective Outcomes

Here we present updated findings of measures reported in our earlier manuscript (Dennehy & Dasgupta, 2017), which include aspirations to pursue a career related to engineering, as well as feelings of belonging and anxiety in engineering.

Engineering Career Aspirations

Participants answered how likely they were to pursue a professional job in engineering on a 7-point scale from 1 (not at all likely) to 7 (very likely)[1]. These items were asked at three time-points during participants' first year in college and subsequently once a year until one-year post-graduation.

Whereas women without a mentor displayed a significant decline in their aspirations to pursue an engineering-related career through college to one-year post-graduation (B = -0.16, SE = 0.08, p = .048, power = .80), those with male mentors (B = -0.07, SE = 0.08, p = .353) and female mentors (B = -0.10, SE = 0.07, p = .180) remained steady. We interpret these results with caution because the change trajectories of the three conditions did not significantly differ from each other (female mentor vs. no mentor, B = 0.07, SE = 0.11, p = .539; male mentor vs. no mentor, B = 0.09, SE = 0.11, p = .403; female mentor vs. male mentor, B = 0.03, SE = 0.10, p = .810).

Belonging

Participants completed four items[2] measuring how much they felt they belonged in engineering: "I feel connected to my peers in engineering;" "I feel accepted by my peers in engineering;" "I feel like an outsider among my peers in engineering" (reverse coded); and "I feel invisible among my peers in engineering" (reverse coded). Participants who completed

surveys after switching majors and graduation completed the items in the past tense referring to their experience in the most recent engineering classes they took (e.g., “I felt connected to my peers in engineering”). Participants indicated how much they agreed with each statement from 1 (not at all true) to 7 (very true). Responses were averaged to create an index of belonging (αs between 0.72-0.88). These items were asked at three time-points during participants’ first-year in college and subsequently once a year until one-year post-graduation.

Women’s feelings of belonging in each of the three conditions did not show a significant linear change when the entirety of the college experience plus one-year post-graduation was examined together (no mentor condition: $B = -0.08$, $SE = 0.05$, $p = .092$; male mentor condition: $B = -0.06$, $SE = 0.04$, $p = .144$; female mentor condition: $B = 0.01$, $SE = 0.04$, $p = .887$).

Anxiety

Drawing from previous research[3-6], the five items that measured anxiety were: “My engineering related classes this year are likely to be difficult;” “I feel worried about my engineering related classes this year;” “I feel stressed about my engineering related classes this year;” “I feel unsure about my engineering related classes this year;” and “I feel anxious about my engineering related classes this year.” If participants had switched out of engineering or graduated, they were asked to think about the last few classes they had taken and to answer how they had felt (e.g., “I felt worried about my engineering related classes”). Participants reported how much they agreed with each statement on a scale from 1 (not at all true) to 7 (very true). Responses to these items were averaged to form an index of anxiety (αs between 0.83-0.90). These items were asked at three time-points during participants’ first-year in college and subsequently once a year until one-year post-graduation.

Women's feelings of anxiety in engineering also did not show a significant linear change when the entirety of the college experience plus one-year post-graduation was considered together (no mentor condition: $B = 0.04$, $SE = 0.05$, $p = .417$; male mentor condition: $B = 0.08$, $SE = 0.05$, $p = .103$; female mentor condition: $B = 0.05$, $SE = 0.05$, $p = .307$).

Testing for Mentor Nonindependence

Given that mentors often had more than one mentee, as we had 58 mentors and 150 mentees, we also conducted three-level unconditional models to identify whether specific mentors played a role in our academic outcomes. In each model, which was conducted for each continuous variable, level 1 was within participants capturing variability across time, level 2 accounted for individual differences between participants (i.e., each participant's ID), while level 3 tested for differences between individual mentors (i.e., each mentor cluster's ID). Intraclass correlations, which are the ratios of the variance in a given level (in this case, the mentor cluster ID variable) relative to the total variance in participants' responses, were conducted. The intraclass correlations for our mentor cluster variable were small (ICCs < 0.08) and the variances were all nonsignificant (ps > .25), meaning that very little variance in participants' responses on each dependent variable is attributed to having a common mentor.

Table S1

Intraclass Correlations and Variance Attributed to Individual Mentors

Variable	ICC for Mentor Cluster	Variance of Mentor Cluster
Anxiety	0.08	$B = 0.12$, $p = .251$
Belonging	0.06	$B = 0.08$, $p = .481$
Confidence	0.06	$B = 0.08$, $p = .475$
Motivation	0.06	$B = 0.05$, $p = .493$
Graduate Intentions	0.05	$B = 0.11$, $p = .724$
Career Intentions	0.06	$B = 0.10$, $p = .449$
Emotional Well-being	0.08	$B = 0.15$, $p = .547$

Table S1 shows intraclass correlations and variance estimates attributed to individual mentor clusters, which were calculated through three-level unconditional models. All significance tests are two-tailed.

Mean Differences at Baseline

To ensure the success of our random assignment, we list condition means at baseline below and whether there were significant differences between conditions.

Table S2

Condition Means at Baseline

	No Mentor	Male Mentor	Female Mentor
	Mean (*SE*)	Mean (*SE*)	Mean (*SE*)
Anxiety	4.55 (0.15)	4.03 (0.17)	4.32 (0.20)
Belonging	5.41 (0.17)	5.40 (0.15)	5.19 (0.16)
Confidence	5.23 (0.13)	5.17 (0.13)	4.92 (0.13)
Motivation	5.49 (0.14)	5.56 (0.12)	5.42 (0.15)
Graduate Intentions	5.85 (0.17)	5.57 (0.19)	5.06 (0.22)[a]
Career Intentions	6.32 (0.13)	6.39 (0.11)	6.10 (0.20)
Emotional Well-being	4.89 (0.23)	5.00 (0.23)	4.81 (0.22)

[a] Denotes that comparison with no mentor condition is $p < .05$

Table S2 presents condition means at baseline (before conditions were assigned). Mean differences were calculated using one-way ANOVAs (two-tailed) with Bonferroni corrections for condition comparisons.

Supplementary References

[1] Stout, J. G., Dasgupta, N., Hunsinger, M., & McManus, M. A. (2011). STEMing the tide: using ingroup experts to inoculate women's self-concept in science, technology, engineering, and mathematics (STEM). *Journal of Personality and Social Psychology*, *100*(2), 255-270.

[2] Good, C., Rattan, A., & Dweck, C. S. (2012). Why do women opt out? Sense of belonging and women's representation in mathematics. *Journal of Personality and Social Psychology*, *102*(4), 700-717.

[3] Beltzer, M. L., Nock, M. K., Peters, B. J., & Jamieson, J. P. (2014). Rethinking butterflies: The affective, physiological, and performance effects of reappraising arousal during social evaluation. *Emotion*, *14*(4), 761.

[4] Harvey, A., Nathens, A. B., Bandiera, G., & LeBlanc, V. R. (2010). Threat and challenge: cognitive appraisal and stress responses in simulated trauma resuscitations. *Medical Education*, *44*(6), 587-594.

[5] Mendes, W. B., Blascovich, J., Major, B., & Seery, M. (2001). Challenge and threat responses during downward and upward social comparisons. *European Journal of Social Psychology*, *31*(5), 477-497.

[6] Moore, L. J., Vine, S. J., Wilson, M. R., & Freeman, P. (2012). The effect of challenge and threat states on performance: An examination of potential mechanisms. *Psychophysiology*, *49*(10), 1417-1425.
