# Supplementary material for: Female peer mentors early in college have lasting positive impacts on female engineering students that persist beyond graduation
Source: Nat Commun. 2022 Nov 11;13:6837. doi: 10.1038/s41467-022-34508-x (PMC9652302; doi:10.1038/s41467-022-34508-x)
Supplement: Supplementary file 3 — Reporting Summary [file 41467_2022_34508_MOESM3_ESM.pdf]

nature portfolio

Corresponding author(s): Nilanjana Dasgupta
Last updated by author(s): Oct 6, 2022

Reporting Summary

Nature Portfolio wishes to improve the reproducibility of the work that we publish. This form provides structure for consistency and transparency in reporting. For further information on Nature Portfolio policies, see our Editorial Policies and the Editorial Policy Checklist.

Statistics

For all statistical analyses, confirm that the following items are present in the figure legend, table legend, main text, or Methods section.

n/a	Confirmed	
☐	☒	The exact sample size (n) for each experimental group/condition, given as a discrete number and unit of measurement
☐	☒	A statement on whether measurements were taken from distinct samples or whether the same sample was measured repeatedly
☐	☒	The statistical test(s) used AND whether they are one- or two-sided<br>*Only common tests should be described solely by name; describe more complex techniques in the Methods section.*
☒	☐	A description of all covariates tested
☒	☐	A description of any assumptions or corrections, such as tests of normality and adjustment for multiple comparisons
☐	☒	A full description of the statistical parameters including central tendency (e.g. means) or other basic estimates (e.g. regression coefficient) AND variation (e.g. standard deviation) or associated estimates of uncertainty (e.g. confidence intervals)
☐	☒	For null hypothesis testing, the test statistic (e.g. F, t, r) with confidence intervals, effect sizes, degrees of freedom and P value noted<br>*Give P values as exact values whenever suitable.*
☒	☐	For Bayesian analysis, information on the choice of priors and Markov chain Monte Carlo settings
☐	☒	For hierarchical and complex designs, identification of the appropriate level for tests and full reporting of outcomes
☒	☐	Estimates of effect sizes (e.g. Cohen's d, Pearson's r), indicating how they were calculated

Our web collection on statistics for biologists contains articles on many of the points above.

Software and code

Policy information about availability of computer code

Data collection	Qualtrics
Data analysis	Mplus 8, SPSS 26

For manuscripts utilizing custom algorithms or software that are central to the research but not yet described in published literature, software must be made available to editors and reviewers. We strongly encourage code deposition in a community repository (e.g. GitHub). See the Nature Portfolio guidelines for submitting code & software for further information.

Data

Policy information about availability of data

All manuscripts must include a data availability statement. This statement should provide the following information, where applicable:

- Accession codes, unique identifiers, or web links for publicly available datasets
- A description of any restrictions on data availability
- For clinical datasets or third party data, please ensure that the statement adheres to our policy

Data and syntax for each model in this manuscript can be found at https://osf.io/968ta/.

Human research participants

Policy information about studies involving human research participants and Sex and Gender in Research.

Reporting on sex and gender	Our findings are focused on sex. We tested the benefits of female and male mentors for female mentees. All participants were female, and all self-reported as such.
Population characteristics	See above.
Recruitment	All female students in their first year of engineering at the University of Massachusetts Amherst were invited to take part in the study for four consecutive cohorts. They were told that the study was examining student experiences in engineering majors and were unaware that the experiment was studying the effects of mentoring. Because participants voluntarily opted into the experiment, recruitment may have been affected by selection bias such that those who decided to participate may have been more committed to engineering than those who declined to participate. However, once participants opted in, they were randomly assigned to one of three conditions with no personal choice, which protects internal validity of the experiment and causal inference of the results.
Ethics oversight	University of Massachusetts Amherst Institutional Review Board

Note that full information on the approval of the study protocol must also be provided in the manuscript.

Field-specific reporting

Please select the one below that is the best fit for your research. If you are not sure, read the appropriate sections before making your selection.

☐ Life sciences ☒ Behavioural & social sciences ☐ Ecological, evolutionary & environmental sciences

For a reference copy of the document with all sections, see nature.com/documents/nr-reporting-summary-flat.pdf

Behavioural & social sciences study design

All studies must disclose on these points even when the disclosure is negative.

Study description	This study is a quantitative, longitudinal, random-controlled experiment.
Research sample	The participants are female engineering students at the University of Massachusetts Amherst. This sample is representative of female engineering students at a predominantly white, public research university. We chose this study sample because we are interested in whether same-sex peer mentors increase persistence and success of female engineering students in disciplines where they are underrepresented; what outcomes are most impacted; and how long these benefits last. The corresponding author was awarded a grant from the National Science Foundation to examine student success at this particular university.
Sampling strategy	No a priori power analysis was conducted. Data were collected from 4 consecutive cohorts of women students entering engineering during a 4-year grant funded period. Every effort was made to recruit the maximum number of first-year women students available in each cohort. On average, we were able to recruit 45%-65% of women in each entering cohort during the 4-year period. Based on Monte Carlo power simulations, we find that most of our significant effects were well-powered (i.e., over 80% power).
Data collection	For the surveys taken in the first year (while mentorship was active), participants took each survey on laptops in a quiet location where they were alone. The research assistant who administered the surveys were blind to participants' condition. Each follow-up assessment after the first year was administered through an online survey portal.
Timing	Data collection began in the fall of 2011 and ended in the spring of 2019. Four separate cohorts of students (2011-12, 2012-13, 2013-14, 2014-15) completed the survey 3 times (beginning, middle, and end of year) during their first year and completed it once each year until one year after graduation.
Data exclusions	No data was excluded.
Non-participation	Our overall response rate was 74%.
Randomization	Participants were randomly assigned to one of three groups. They were either assigned no mentor, a male mentor, or a female mentor.

Reporting for specific materials, systems and methods

We require information from authors about some types of materials, experimental systems and methods used in many studies. Here, indicate whether each material, system or method listed is relevant to your study. If you are not sure if a list item applies to your research, read the appropriate section before selecting a response.

Materials & experimental systems

n/a	Involved in the study
☒	☐ Antibodies
☒	☐ Eukaryotic cell lines
☒	☐ Palaeontology and archaeology
☒	☐ Animals and other organisms
☒	☐ Clinical data
☒	☐ Dual use research of concern

Methods

n/a	Involved in the study
☒	☐ ChIP-seq
☒	☐ Flow cytometry
☒	☐ MRI-based neuroimaging
